# Implementing the Personalised Exercise Rehabilitation in Cancer Survivorship (PERCS) Triage and Referral System in Ireland: Protocol for a Qualitative Stakeholder Study

**DOI:** 10.12688/hrbopenres.14272.2

**Published:** 2026-03-10

**Authors:** Marie Tierney, Gráinne Sheill, Elaine Toomey, Dearbhaile O'Hare, Tom Conway, Louise Mullen, Claire L Donohoe, Emer Guinan

**Affiliations:** 1 Trinity St James's Cancer Institute, Dublin, Ireland; 2 Discipline of Physiotherapy, School of Medicine, Trinity College Dublin, Dublin, Ireland; 3 Department of Physiotherapy, St James's Hospital, Dublin, Ireland; 4 Centre for Health Research Methodology, School of Nursing & Midwifery, University of Galway, Galway, Ireland; 5 Institute for Clinical Trials, College of Medicine, Nursing & Health Sciences, University of Galway, Galway, Ireland; 6 Independent Public and Patient Involvement Contributor, Ireland, Ireland; 7 National Cancer Control Programme, Health Service Executive, Dublin, Ireland; 8 Department of Surgery, St James's Hospital, Dublin, Ireland

**Keywords:** Exercise rehabilitation, Cancer survivorship, PERCS, National implementation, Barriers and facilitators, Qualitative study

## Abstract

Background

Exercise rehabilitation offers substantial benefits for people living with and beyond cancer, improving physical, psychological, and disease-related outcomes. Despite strong evidence and policy support, integration of exercise into routine cancer care in Ireland remains limited. The PERCS (Personalised Exercise Rehabilitation in Cancer Survivorship) triage and referral system was developed to provide a structured, stepped-care model directing patients to the appropriate level of exercise support. Following on from a feasibility study, this protocol describes a qualitative study to inform national implementation.

Methods

This multi-stakeholder study will use focus groups and interviews with four stakeholder groups: 1) Healthcare professionals, 2) Exercise professionals, 3) Policymakers, charity partners, and cancer centre managers, and 4) People living with and beyond cancer, carers and family members. Topic guides, informed by the Consolidated Framework for Implementation Research (CFIR) and tailored to each stakeholder group, will seek to explore barriers, facilitators, and contextual factors that influence implementation. Data will be analysed using framework analysis. Transcripts will be coded using both a CFIR-based deductive coding approach and inductive codes that are relevant to the research aim, and key points will be organised to allow comparison of responses across participant groups. The findings stemming from this work will inform the selection of tailored implementation strategies mapped to the Expert Recommendations for Implementing Change (ERIC) taxonomy.

Conclusion

This study will generate detailed, context-sensitive evidence on the barriers and facilitators to implementing the PERCS system at a national level. By engaging with key stakeholders and using a structured implementation science approach, the findings will guide the development of practical, policy-relevant strategies to support the integration of exercise rehabilitation into survivorship care. The study will contribute to ongoing efforts to improve quality of life for cancer survivors in Ireland and build capacity for sustainable, person-centred cancer rehabilitation services.

## Introduction

Over 200,000 people in Ireland are currently living with and beyond cancer, and this number is projected to rise significantly as 10-year survival rates continue to improve, in line with European estimates ( Lawler *et al.*, 2023). With survivorship increasing, there is growing recognition of the importance of addressing the longer-term health and quality-of-life needs of this population. The National Cancer Strategy 2017–2026 ( Department of Health, 2017) explicitly commits to enhancing quality of life for survivors, recognising exercise rehabilitation as a key component of survivorship care.

Cancer treatment can result in a range of physical and psychosocial sequelae, including fatigue, muscle weakness, reduced cardiopulmonary fitness, anxiety, and depression, many of which are amenable to targeted exercise interventions ( Campbell *et al.*, 2019; Courneya *et al.*, 2025). An estimated 60–90% of individuals affected by cancer have at least one specialised rehabilitation need ( Pergolotti *et al.*, 2015), which may include cancer-related comorbidities or impairments such as weakness, cachexia, loss of balance, lymphoedema, or pain. There is robust international evidence supporting the role of exercise in improving physical, psychological, and disease-related outcomes, including fatigue, functional capacity, and health-related quality of life, for people living with and beyond cancer but it is important that exercise support is adaptable to individual needs. Patients with significant impairments may benefit from hospital- or primary-care-based rehabilitation programmes delivered by rehabilitation specialists, while physically inactive patients without impairments may be well served by community-based exercise programmes delivered by physiotherapists or exercise specialists ( Coletta *et al.*, 2020; Schmitz *et al.*, 2021).

To allow translation of exercise guidelines into practice, coordinated systems are needed to direct patients to the appropriate level of care, balancing clinical need with service capacity. This typically involves collaborative models that span hospital and community settings. Internationally, stepped-care approaches and triage and referral systems have been proposed to guide decision-making and streamline access to appropriate supports ( Capozzi *et al.*, 2023; Covington *et al.*, 2021; Dennett *et al.*, 2020; Mina *et al.*, 2018; Schmitz *et al.*, 2024; Stout *et al.*, 2020). These models generally recognise a spectrum of complexity arising from comorbidities and treatment side-effects, incorporate screening assessments to identify risks requiring medical or rehabilitation oversight and recommend tailored referrals to supervised rehabilitation, community exercise programmes, or self-management supports. While some of these frameworks are underpinned by formal triage processes ( Capozzi *et al.*, 2023; Covington *et al.*, 2021; Stout *et al.*, 2020), most remain theoretical. Only a limited number have been tested for feasibility and implementation in real-world settings, demonstrating positive outcomes ( Capozzi *et al.*, 2023; Schmitz *et al.*, 2024). These models also typically operate within health systems such as those in the United States, Canada, and Australia where access to cancer physiatry, clinical exercise physiologists, and tiered rehabilitation services are well established.

In contrast, the vision of every cancer patient in Ireland having access to exercise during and after treatment is not yet being realised. Survivors often face multiple barriers to engagement, including a lack of professional guidance, unclear referral pathways, and limited local service availability ( Brennan *et al.*, 2022; Nadler *et al.*, 2017; Riordan *et al.*, 2025). Ireland does not currently have a recognised cancer physiatry discipline, and clinical exercise physiologists are not formally integrated into the healthcare system. Rehabilitation services remain unevenly distributed and poorly resourced, contributing to inconsistent access to appropriate support.

To address this gap, the research team developed the Personalised Exercise Rehabilitation in Cancer Survivorship (PERCS) system, a triage and referral model tailored to the Irish healthcare context. The system assesses cancer survivors’ exercise needs and guides referral to one of three appropriate pathways: (1) independent exercise with provision of online resources, (2) community-based exercise programmes, or (3) physiotherapist-led rehabilitation.

PERCS 1.0, funded by the Irish Cancer Society, tested the feasibility and acceptability of this system through a physiotherapy-led clinic for survivors of cancer treated during the COVID-19 pandemic. Results were highly encouraging and demonstrated the feasibility of a structured triage pathway to support exercise participation across differing levels of need:

• Over 90% of participants required support to become physically active ( Brennan *et al.*, 2024).
• Triage referred 9.4% of participants to **Level 1**, where advice and encouragement were provided to support independent physical activity and self-management. The majority (67.2%) were referred to **Level 2** community-based supervised exercise programmes, delivered either in person or online according to individual needs and preferences. A further 23.4% were allocated to **Level 3**, involving referral to physiotherapy-led rehabilitation services to address more complex rehabilitation needs ( Brennan *et al.*, 2024).
• Adherence to physical activity guidelines improved for both aerobic and resistance exercise at 12 weeks ( Brennan *et al.*, 2024).
• Participants reported high satisfaction with the personalised assessment and referral approach ( Brennan *et al.*, 2025).

These findings provided critical proof-of-concept for the PERCS triage and referral approach, demonstrating real-world feasibility and positive patient impact within the Irish setting. PERCS 2.0 seeks to build on this work by shifting the focus from local feasibility to national implementation through the exploration of and response to the contextual factors influencing the scaling of the PERCS system across the Irish healthcare landscape.

## Aims and objectives

The aim of this study is to inform the national implementation of the PERCS triage and referral system by understanding key contextual factors influencing its uptake and identifying strategies to support its successful delivery across the Irish healthcare system.

To achieve this aim, the objectives are:

• To explore the contextual factors influencing the implementation of the PERCS triage and referral system nationally and.
• To identify and prioritise the implementation strategies that can address these contextual factors.

These aims will be achieved using the Consolidated Framework for Implementation Research (CFIR) to guide data collection and analysis and the Expert Recommendations for Implementing Change (ERIC) taxonomy to map factors to potential strategies.

## Methods

### Study design

This study will utilise a qualitative methodology, collecting data through focus groups and individual interviews. Both formats will follow the same semi-structured topic guide and the choice of formats is offered to ensure flexibility and accessibility for participants. Focus groups will be used where appropriate to explore shared perspectives and group dynamics, while interviews will be conducted with individuals who prefer or require a one-on-one format (e.g. due to time constraints, or geographic location). The use of both formats is not intended to produce different types of responses, but rather to maximise inclusivity and engagement across a diverse range of stakeholder groups.

### Sample selection and recruitment

A purposive sampling strategy will be used to ensure maximum variation across roles, geography, and healthcare settings. Four stakeholder groups will be recruited:

1. Healthcare professionals involved in cancer care (e.g. oncology nurses, consultants, physiotherapists).
2. Exercise professionals.
3. Policymakers, charity partners, and cancer centre managers.
4. People living with and beyond cancer, carers, and family members.

Inclusion criteria include adults aged 18 or older with direct experience relevant to one of the stakeholder groups. Participants must be able to provide informed consent and take part in English.

Participants will be identified through project partner organisations, clinical contacts, patient networks, and the research team’s stakeholder database. Potential participants will be informed about the study through a recruitment letter/poster/social media post outlining essential information about the study. Individuals interested in learning more about the study will be encouraged to contact the lead investigator directly to receive further information via a participant information leaflet (Appendix 1), ask questions, and discuss any aspect before deciding whether to participate.

### Sample size

We anticipate recruiting approximately 50 participants across stakeholder groups, though the final number may vary. This sample was chosen to ensure sufficient diversity across participant groups and to enable comprehensive charting with the Framework Analysis approach.

### Intervention context and analytical approach

The intervention under study is the PERCS triage and referral system, which comprises three stages:

1. Patient Assessment
2. Triage
3. Creation of a personalised exercise plan

Participants will receive a brief description and/or infographic of the PERCS model before or at the start of the session to support shared understanding. Data collection and analysis will be guided by CFIR to identify key contextual factors influencing national implementation of the PERCS system. Findings will also inform the development of relevant, stakeholder-informed implementation strategies, mapped to the ERIC taxonomy.

### Data collection and analysis

All data collection will be conducted by an experienced qualitative researcher trained in implementation science. Data will be collected via focus groups and individual interviews using semi-structured topic guides tailored to four stakeholder groups: healthcare professionals, exercise professionals, policymakers/cancer centre managers/charity representatives, and people living with and beyond cancer, carers and family members.

The topic guides (Appendix 2) were developed collaboratively by the research team using CFIR to highlight relevant domains and constructs for exploring contextual influences on the implementation of the PERCS triage and referral system. Development was also informed by findings from the PERCS 1.0 feasibility study and input from both the research team and Public and Patient Involvement (PPI) panel.

Initially draft guides were created and tailored to each stakeholder group but maintained a common structure to ensure comparability. They were reviewed by experts in implementation science, clinical stakeholders, and PPI contributors, and refined though an iterative process until agreement was reached. Each guide includes prompts on awareness of the PERCS model, anticipated barriers and enablers, relevance to local context, and support needs for embedding the model in routine care.

All interviews and focus groups will be audio-recorded, transcribed verbatim, and pseudonymised. Transcripts will be analysed using framework analysis, an approach suited to applied research using structured conceptual frameworks like CFIR.

Analysis will proceed through the following steps:

• Familiarisation: The researcher will read and re-read transcripts to immerse themselves in the data.
• Coding: A coding framework will be developed based on CFIR constructs, supplemented by inductive codes that emerge during analysis. This hybrid approach allows for both theoretical alignment and responsiveness to stakeholder perspectives. One researcher will code the transcripts, with another independently reviewing a sample of the transcripts to support rigour. There will also be regular team discussions to support reflexivity, enhance consistency, and ensure that interpretations remain grounded in the data.
• Indexing: The framework will be applied systematically across transcripts.
• Charting: Key data will be summarised in a matrix by code and participant group to enable comparison across settings, roles, and perspectives.
• Mapping and interpretation: Emerging patterns will be interpreted within and across CFIR domains to identify key contextual influences on implementation. Findings will be used to inform a set of prioritised implementation strategies, mapped to the ERIC taxonomy.

All data management and analysis will be supported using NVivo software.

### Ethical considerations

Ethical approval has been obtained from the TCD School of Medicine Research Ethics Committee (approval number: 250606). All participants will receive a detailed Participant Information Leaflet and provide written informed consent prior to participation.

Data will be pseudonymised and stored securely on encrypted university servers, in compliance with GDPR and HRB data governance policies. Transcripts will be anonymised, and any direct identifiers removed before analysis.

### Dissemination plan

• Findings from this study will be disseminated through:
• Peer-reviewed journal articles and national/international conference presentations
• Stakeholder workshops and briefing documents tailored to HSE, cancer centres, and community partners
• Lay summaries for participants and the broader cancer survivor community

All outputs will align with HRB Open Research policies for transparency, accessibility, and research integrity.

## Conclusion

This study aims to generate in-depth, contextually relevant information which can feed into the implementation of the PERCS triage and referral system for exercise rehabilitation in cancer survivorship across Ireland. By engaging with a broad range of stakeholders and applying the CFIR framework within a rigorous qualitative methodology, the research will identify key barriers, facilitators, and contextual considerations that influence implementation. These findings will inform the development of targeted, stakeholder-informed strategies to support the national scale-up of PERCS and contribute to broader efforts to integrate exercise into routine cancer care. The study will provide a valuable platform for future implementation planning, policy development, and service design of cancer rehabilitation.

## Data Availability

No data are associated with this article. Open Science Framework. Implementing the Personalised Exercise Rehabilitation in Cancer Survivorship (PERCS) Triage and Referral System in Ireland: Protocol for a Qualitative Stakeholder Study: 10.17605/OSF.IO/RDJYV Extended data in this project pertain to: Appendix 1: Participant Information leaflets Appendix 2: Topic guides for interviews and focus groups Data are available under the terms of the Creative Commons Attribution 4.0 International license (CC-BY 4.0).
