# Supplementary material for: Implementing the Personalised Exercise Rehabilitation in Cancer Survivorship (PERCS) Triage and Referral System in Ireland: Protocol for a Qualitative Stakeholder Study
Source: HRB Open Res. 2026 Mar 10;8:115. Originally published 2025 Oct 27. [Version 2] doi: 10.12688/hrbopenres.14272.2 (PMC13109707; doi:10.12688/hrbopenres.14272.2)
Supplement: Peer review history

# Reviewer report: Reviewer response for version 2

**DOI:** 10.21956/hrbopenres.15828.r54104

**Published:** 2026-04-24

**Authors:** Sarah J Hardcastle

**Affiliations:** 1 Sheffield Hallam University, Sheffield, UK

The proposed study contributes to an important area of work within exercise oncology concerning the implementation pathways for physical activity promotion.

 The introduction could be strengthened with a couple of additions: (i) reference to the national and international physical activity guidelines for those living with and beyond cancer (and in doing so, defining what is meant be physically inactive patients) and (ii) reference to the previous research in Ireland in relation to the challenges to physical activity promotion and recommendations for implementation [1,2]. For example, the recently published scoping review of physical activity and exercise initiatives for cancer patients in Ireland for the National Cancer Control Programme and associated (some of this review focused on challenges and solutions has also been published [1]).

 This is highly relevant given the findings on limited access to appropriate physical activity interventions for cancer patients in Ireland, and also to poor uptake to and high attrition to cancer specific community-based exercise programme .

Is the study design appropriate for the research question?

Yes

Is the rationale for, and objectives of, the study clearly described?

Yes

Are sufficient details of the methods provided to allow replication by others?

Yes

Are the datasets clearly presented in a useable and accessible format?

Not applicable

Reviewer Expertise:

health behaviour change, exercise and health psychology, adherence, exercise oncology.

I confirm that I have read this submission and believe that I have an appropriate level of expertise to confirm that it is of an acceptable scientific standard.

## References

[1] : Promoting Physical Activity to Cancer Survivors in Practice: Challenges and Solutions for Implementation. *Cancers* .2025;17(5) : 10.3390/cancers17050850 10.3390/cancers17050850 PMC1189890240075697

[2] : Healthcare professionals' knowledge and practice of physical activity promotion in cancer care: Challenges and solutions. *European Journal of Cancer Care* .2018;27(2) : 10.1111/ecc.12795 10.1111/ecc.12795 29193416

# Reviewer report: Reviewer response for version 1

**DOI:** 10.21956/hrbopenres.15700.r51858

**Published:** 2025-12-12

**Authors:** Francesca Rossi

**Affiliations:** 1 Regina Margherita Children’s Hospital, Turin, Italy

The study design is correctly identified in the title. The background, aims, objectives and methods of the study are all crearly described.

 However, I have a few suggestions to help the authors improve their paper:

 Abstract:

 In the conclusion section of the abstract, you assert that the study “will contribute to ongoing efforts to improve quality of life for cancer survivors”. However, you do not mention the impact of exercise on quality of life anywhere else in the text. You should include this information in the introduction, where you discuss the role of exercise.

 Introduction:

 You refer to the results of the PERCS 1.0 for the majority of your sample, which consisted of patients requiring support to become physically active and to those who were referred to community exercise programmes. However, these are incomplete data. It would also be interesting for the reader to know also the percentage of patients who were referred to independent exercise programmes and to physiotherapist-led rehabilitation programmes.

 Methods:

 You should explain what the acronym “PPI” stands for the first time you mention it.

Is the study design appropriate for the research question?

Yes

Is the rationale for, and objectives of, the study clearly described?

Yes

Are sufficient details of the methods provided to allow replication by others?

Yes

Are the datasets clearly presented in a useable and accessible format?

Not applicable

Reviewer Expertise:

pediatric oncology rehabilitation

I confirm that I have read this submission and believe that I have an appropriate level of expertise to confirm that it is of an acceptable scientific standard.
